# Purine nucleoside phosphorylase deficiency induces p53-mediated intrinsic apoptosis in human induced pluripotent stem cell-derived neurons

**DOI:** 10.1038/s41598-022-10935-0

**Published:** 2022-05-31

**Authors:** Michael Tsui, Jeremy Biro, Jonathan Chan, Weixian Min, Kerry Dobbs, Luigi D. Notarangelo, Eyal Grunebaum

**Affiliations:** 1 Developmental and Stem Cell Biology Program, Hospital for Sick Children, Toronto, ON Canada; 2 The Institute of Medical Sciences, The University to Toronto, Toronto, ON Canada; 3 Laboratory of Clinical Immunology and Microbiology, Division of Intramural Research, National Institute of Allergy and Infectious Diseases, National Institutes of Health, Bethesda, USA; 4 Division of Immunology and Allergy, Department of Pediatrics, The Hospital for Sick Children, 555 University Avenue, Toronto, ON M5G1X8 Canada

**Keywords:** Cell biology, Immunology

## Abstract

Purine nucleoside phosphorylase (PNP) is an important enzyme in the purine degradation and salvage pathway. PNP deficiency results in marked T lineage lymphopenia and severe immunodeficiency. Additionally, PNP-deficient patients and mice suffer from diverse non-infectious neurological abnormalities of unknown etiology. To further investigate the cause for these neurologic abnormalities, induced pluripotent stem cells (iPSC) from two PNP-deficient patients were differentiated into neurons. The iPSC-derived PNP-deficient neurons had significantly reduced soma and nuclei volumes. The PNP-deficient neurons demonstrated increased spontaneous and staurosporine-induced apoptosis, measured by cleaved caspase-3 expression, together with decreased mitochondrial membrane potential and increased cleaved caspase-9 expression, indicative of enhanced intrinsic apoptosis. Greater expression of tumor protein p53 was also observed in these neurons, and inhibition of p53 using pifithrin-α prevented the apoptosis. Importantly, treatment of the iPSC-derived PNP-deficient neurons with exogenous PNP enzyme alleviated the apoptosis. Inhibition of ribonucleotide reductase (RNR) in iPSC derived from PNP-proficient neurons with hydroxyurea or with nicotinamide and trichostatin A increased the intrinsic neuronal apoptosis, implicating RNR dysfunction as the potential mechanism for the damage caused by PNP deficiency. The findings presented here establish a potential mechanism for the neurological defects observed in PNP-deficient patients and reinforce the critical role that PNP has for neuronal viability.

## Introduction

Purine nucleoside phosphorylase (PNP) is an evolutionarily conserved enzyme important for purine degradation and salvage. PNP is responsible for the phosphorylation of inosine and deoxyinosine into hypoxanthine, as well as guanosine (Guo) and deoxyguanosine (dGuo) into guanine, which are eventually converted into uric acid. Alternatively, PNP substrates can be utilized for the generation of deoxynucleotide triphosphates (dNTP), including deoxyguanosine triphosphate (dGTP).

Bi-allelic defects in the *PNP* gene result in the accumulation of the enzyme’s substrates, including dGuo that has been shown to be toxic to cells. PNP enzyme is ubiquitously expressed, yet the highest activity is in lymphoid cells, explaining in part the preferential effects of impaired PNP activity on the immune system, particularly thymocytes and T cells. Indeed, PNP-deficient patients display marked lymphopenia, profound and progressive T lineage immunodeficiency as well as increased susceptibility to infections, autoimmunity, and malignancy that are often lethal in childhood^1^. Previous studies have attributed the T lineage abnormalities to the accumulation of the PNP substrate dGuo that can be phosphorylated by the mitochondrial deoxyguanosine kinase (dGK) enzyme to dGTP. The accumulation of dGTP in thymocytes can cause mitochondrial-membrane potential (MMP) reduction and cytochrome c release, indicative of mitochondrial disruption and intrinsic apoptosis^2^. It has also been hypothesized that dNTP imbalance in PNP deficiency can lead to allosteric inhibition of ribonucleoside reductase (RNR), which is responsible for the de novo synthesis of deoxyribonucleotides, necessary for DNA replication and repair^3,4^. Additionally, chemical inhibition of PNP induced apoptosis of patients’ lymphocytes, which was associated with DNA damage-induced tumor suppressor protein p53^5^. Moreover, inhibition of RNR using hydroxyurea (HU) has been shown to cause p53 accumulation^6^ and p53-mediated apoptosis^7^. Similarly, in another purine defect, adenosine deaminase deficiency, thymocyte apoptosis was dependent on p53^8^.

While the immune abnormalities have been considered a hallmark of PNP deficiency, more than 50% of PNP-deficient patients suffer from diverse neurologic defects, manifesting often as motor deficits including spastic paresis or cerebellar ataxia^9,10^. The neurologic abnormalities frequently precede the immune defects, suggesting that they are caused by a non-immune mechanism^11^. Yet, it has been difficult to decipher the pathophysiology of the neurological deficits in PNP-deficient patients due to the limited availability of samples as well as the ethical concerns of obtaining brain tissue. An alternative strategy has been to use relevant animal models. Mice lacking PNP enzyme activity (PNP-KO) suffer from metabolic and immune abnormalities reminiscent of those observed in PNP-deficient patients^3^. PNP-KO mice also display diverse motor-neuron defects, and histological analysis of their brain demonstrated a reduced number of cerebellar Purkinje cells that were malformed and apoptotic^12^. Moreover, treating PNP-KO mice from an early age with PNP enzyme replacement, produced by fusion of the human PNP to the HIV-TAT protein transduction domain (TAT-PNP), prevented the development of neurological abnormalities^12^. Nevertheless, the correlation between the murine findings and the human disease has been debated. Interestingly, akin to the observation in PNP-KO mice^12^, treatment of rats with HU led to a marked reduction in the number and diameter of neurons^13^. HU administration in mice caused p53-mediated apoptosis in neuroepithelial cells^7^, and decreased cerebellum areas and Purkinje cell numbers^14^. Together, this data suggests that RNR inhibition and p53 activation might also be involved in the neuronal damage caused by PNP deficiency.

The ability to differentiate induced pluripotent stem cells (iPSC) into diverse cells has provided an important tool to understand and treat a growing list of human ailments. This strategy has been particularly attractive for studying inherited and acquired neuronal disorders^15–17^. Preliminary work showed previously that iPSC-derived neurons from a PNP-deficient patient had reduced soma and nuclei size^18^. Additionally, iPSC-derived neurons from this patient exhibited increased spontaneous apoptosis.

Here, neurons were generated from the iPSCs of 2 PNP-deficient patients to conclusively demonstrate enhanced intrinsic apoptosis with reduced MMP and increased caspase-9 (casp-9) expression in its cleaved activated form. Furthermore, in these PNP-deficient neurons, p53 expression was elevated, while inhibition of p53 with pifithrin-α (PFT-α) prevented the intrinsic apoptosis, cementing the role of p53-mediated apoptosis in this condition. Furthermore, treating PNP-proficient neurons with the RNR inhibitors HU, or a combination of nicotinamide (NAM) and trichostatin A (TSA)^19^, recapitulated the abnormalities observed in the PNP-deficient neurons, implicating RNR inhibition as a potential contributor to the phenotype observed in PNP-deficient patients. Importantly, restoring purine homeostasis with exogenous PNP corrected the abnormalities observed in PNP-deficient neurons, further supporting the potential use of enzyme replacement therapy for the benefit of patients.

## Methods

### Induced pluripotent stem cells

PNP1^−/−^ iPSC were established from the B lymphoblasts of a patient with compound heterozygous mutations (c.383A>G and c.701G>C) in the PNP gene, as previously described^20^. PNP2^−/−^ iPSC were established from the skin fibroblasts of a 4.5-year-old male with compound heterozygous mutations (c.172C>T, c.89G>A) in the PNP gene, as previously described^21^. PNP1^−/−^ iPSC were generated using the polycistronic lentiviral vector STEMCCA–LoxP with pluripotency demonstrated by expression of OCT4, NANOG, TRA-1-60, TRA-1-81, SSEA3, and SSEA4, as previously described^22^. PNP2^−/−^ iPSC were generated at the Centre for Commercialization of Regenerative Medicine, Toronto, Canada using the non-integrative Sendai virus. B lymphoblasts and skin fibroblasts were similarly used to produce iPSCs from healthy controls (CTL). Pluripotency was confirmed by demonstrating > 97% expression of pluripotency markers OCT4, SOX2, TRA160, and SSEA4. PNP enzyme activity was repeatedly measured in PNP1^−/−^ and PNP2^−/−^ iPSC and confirmed as < 1%, measured by conversion of (^14^C)Inosine, as previously described^23^.

### Neuronal differentiation

Differentiation of iPSCs into neurons was performed as previously described^18^ with minor modification. Briefly, iPSCs were dissociated and suspended in STEMdiff neural progenitor medium (STEMCELL Technologies, 05833, Vancouver BC, Canada) and plated at a density of 2 × 10^6^ cells/well in Aggrewell 800 plates (STEMCELL Technologies, 34811, Vancouver BC, Canada) for 4 days to form embryoid bodies (EB). The EB were then dissociated and plated onto poly-l-ornithine (Sigma-Aldrich, A-004-C, Darmstadt, Germany) and laminin (Sigma-Aldrich, 11243217001, Darmstadt, Germany) coated 6-well plates for an additional 7 days to form neural rosettes. The rosettes were selectively detached using STEMdiff neural rosette selection reagent (STEMCELL Technologies, 05832, Vancouver BC, Canada), suspended in STEMdiff neural induction medium (STEMCELL Technologies, 05835, Vancouver BC, Canada), and plated onto poly-L-ornithine and laminin-coated 6-well plates at a density of 2 × 10^6^ cells/well to generate neural progenitor cells (NPC). The NPCs were then differentiated into neurons by dissociating and plating 1 × 10^5^ cells/well into Falcon chambered cell culture slides (ThermoFisher Scientific, 08-774-25, Waltham MA, USA) using STEMdiff neural induction medium for 14 days. All neurons analyzed in this study are 14 days old, with the exception of the neurons analyzed to determine the rate of apoptosis, whereby analysis was performed daily across days 10–14.

### Characterization of neurons and glial progenitors

To characterize the iPSCs-derived neurons, cells were fixed using 4% paraformaldehyde (ThermoFisher Scientific, 28908, Waltham MA, USA) and permeabilized using 0.2% Igepal CA-630 (Sigma-Aldrich, I3021, Darmstadt, Germany). Neurons were stained with mouse anti-MAP2 (ThermoFisher Scientific, MA5-12826, Waltham MA, USA) and goat anti-mouse Alexa Fluor 488 (ThermoFisher Scientific, A-11001, Waltham MA, USA), or chicken anti-MAP2 (Abcam, ab92434, Cambridge, United Kingdom) and goat anti-chicken Alexa Fluor 488 (ThermoFisher Scientific, A-11039, Waltham MA, USA) to depict their cytoskeletal structure. Nuclei were visualized using the nuclear counterstain DAPI (ThermoFisher Scientific, D1306, Waltham MA, USA). Glial progenitors that were generated from the NPCs were also resolved with mouse anti-MAP2 and goat anti-mouse Alexa Fluor 488 in culture^24^.

### Confocal microscopic imaging

Stained neurons were imaged using Quorum spinning disk confocal microscopes with a 20 × objective magnification, with images captured using the Hamamatsu C9100-13 camera (Quorum Technologies, Guelph ON, Canada). Volocity 6.3 imaging software (Quorum Technologies, Guelph ON, Canada) was used to analyze images. Using the ability to acquire images with a 1 nm z stack, the entire volumes of the cells’ soma and nucleus, rather than a focal plane, were calculated.

### Image analysis

Image analysis was performed by operators unaware of the PNP activity of the cells or treatments, using the Volocity 6.3 imaging software. Fluorescence, reported as relative fluorescence units (RFU), was determined using the Volocity Measure tool in all neurons in the frame. Area, volume, and total RFU were recorded for each parameter. Background RFU was subtracted, and the total RFU was divided by the volume or area to normalize fluorescence values. The analysis of synapse formation was accomplished by counting the number of axon terminals making contact with soma or dendrites.

### Determination of apoptosis

The rate of apoptosis was determined by staining neurons with rabbit anti-cleaved caspase-3 (ThermoFisher Scientific, PA5-114687, Waltham MA, USA) and goat anti-rabbit Alexa Fluor 555 (ThermoFisher Scientific, A-21428, Waltham MA, USA) across days 10–14 of the differentiation to determine the percentage of apoptotic cells over time. Susceptibility of cells to apoptosis was assessed by incubating neurons with 10 µM staurosporine (stau) (Sigma-Aldrich, S5921, Darmstadt, Germany) 1h prior to staining, followed by staining with rabbit anti-cleaved caspase-3 and goat anti-rabbit Alexa Fluor 555. Intrinsic apoptosis was identified by measuring MMP, using MitoTracker Red CMXRos (ThermoFisher Scientific, M7512, Waltham MA, USA) and casp-9 with rabbit anti-cleaved caspase-9 (ThermoFisher Scientific, PA5-17913, Waltham MA, USA) and goat anti-rabbit Alexa Fluor 647 (ThermoFisher Scientific, A-21244, Waltham MA, USA). Expression of p53 was evaluated by staining with mouse anti-p53 (Abcam, ab26, Cambridge, United Kingdom) and goat anti-mouse Alexa Fluor 405 (ThermoFisher Scientific, A-31553, Waltham MA, USA). To further demonstrate that neuronal apoptosis occurs through a p53-mediated pathway, 1μM of the p53 inhibitor PFT-α (Sigma-Aldrich, P4359, Darmstadt, Germany) was added to the differentiation media of PNP-deficient iPSCs for the entirety of the differentiation.

### PNP enzyme supplement treatment

In some experiments, PNP-deficient iPSCs were treated with 1 μM TAT-PNP^25^ that was added to the differentiation media, and replenished with each media change. The concentration of TAT-PNP was chosen as the lowest concentration that improved the viability of PNP-deficient neurons.

### RNR inhibition

To determine similarities between the inhibition of RNR to the effects of PNP deficiency on neuronal development, CTL1 and CTL2 iPSCs were treated with 10μM HU (Sigma-Aldrich, H8627, Darmstadt, Germany), or with 10 mM NAM (Sigma-Aldrich, N0636, Darmstadt, Germany) and 5 μM TSA (Sigma-Aldrich, T8552, Darmstadt, Germany). The concentrations of the RNR inhibitors were chosen as the highest concentrations that did not cause increased apoptosis of undifferentiated iPSCs. Stau (10 μM for 12h) was used as a positive control for HU and NAM + TSA to induce maximal apoptosis.

### Statistical analysis

T-tests, one- and two-way ANOVAs (when appropriate) were used when comparing the cells with or without treatments. Multiple linear regression analysis was utilized using the least-squares model after confirming Gaussian distribution with the D’agostino-Pearson omnibus K2 normality test. A difference of p < 0.05 was considered statistically significant. All p values were identical between the ANOVA and linear regression analysis, unless otherwise stated.

### Ethics statement

All studies involving human participants were reviewed and approved by the Research Ethics Board of the Hospital for Sick Children, Toronto, Ontario, and by the National Institutes of Health Institutional Review Board (protocol 16-I-N139) and in accordance with the Declaration of Helsinki. All studies were performed in accordance with all the relevant guidelines and regulations. The patients/participants and or legal guardians provided their written informed consent to participate in this study.

## Results

### Characterization of iPSC-derived neurons

**Figure 1:**
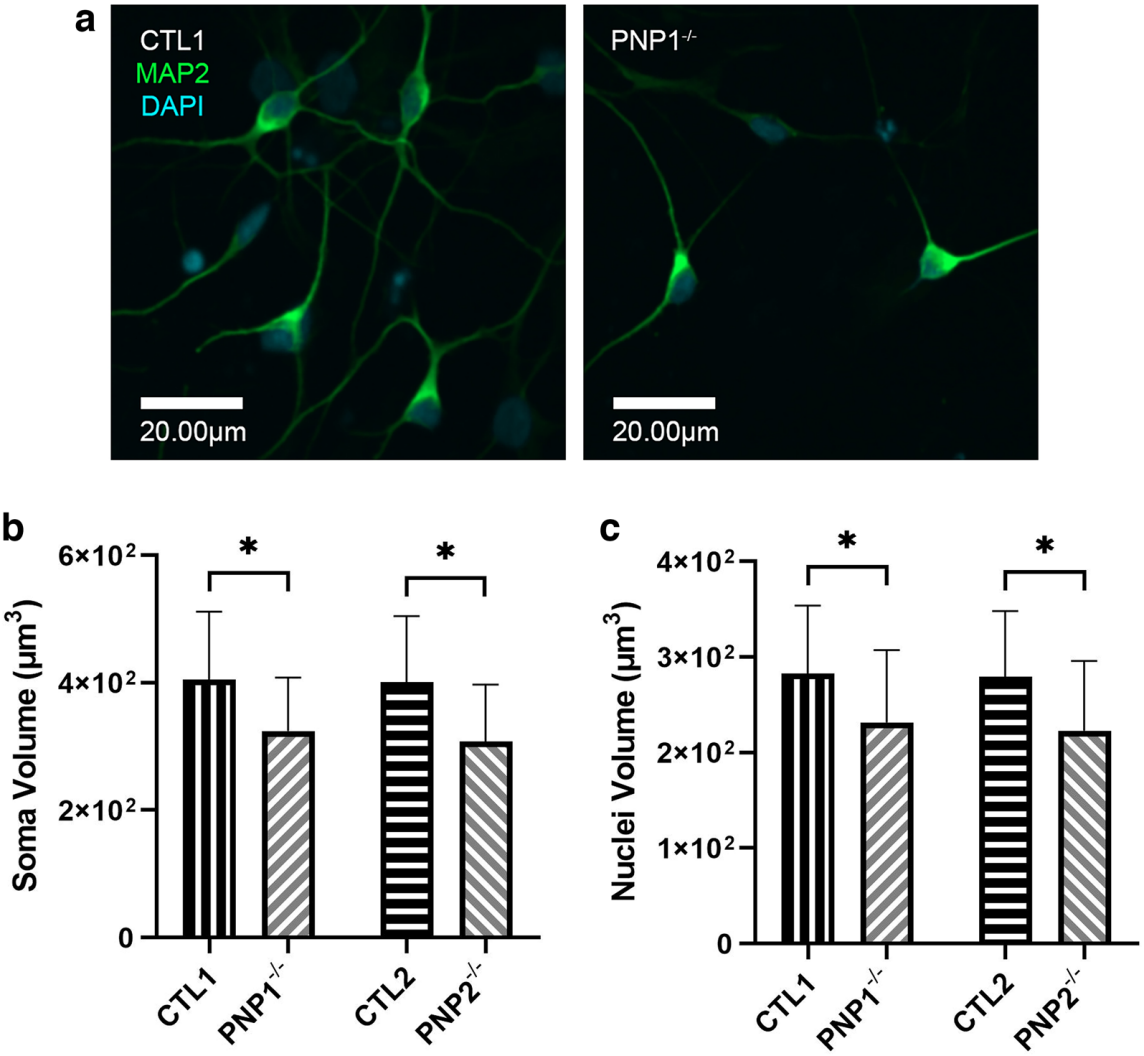
Reduced soma and nuclei volume in PNP-deficient induced pluripotent stem cell-derived neurons. (**A**) Representative immunofluorescence microscopy images depicting the soma and nuclei of neurons derived from control (CTL) and PNP-deficient (PNP^−/−^) iPSCs. The average soma (**B**) and nuclei (**C**) volumes of CTL and PNP^−/−^ neurons. Data are the mean + SD of n = 1050 (350 neurons from 3 replicates); * p < 0.001.

To study the role of PNP in human neurons, iPSCs were established independently from 2 PNP-deficient patients harboring different mutations in the PNP gene and from 2 PNP-proficient healthy controls. The iPSCs were differentiated to EBs and into NPCs, and then into neuronal cells. No differences in neuronal shape were observed between PNP-deficient and PNP-proficient neurons (Fig. 1A). However, the average soma areas of PNP1^−/−^ (p < 0.001) and PNP2^−/−^ (p < 0.001) neurons were significantly reduced in comparison to CTL1 and CTL2 cells, respectively (Supplemental Fig. 1A). Similarly, the average nuclei areas of PNP1^−/−^ (p < 0.001) and PNP2^−/−^ (p < 0.001) neurons were significantly reduced compared to CTL1 and CTL2 cells, respectively (Supplemental Fig. 1B). To avoid potential measurement bias when analyzing single focal planes, fluorescence across the entire volume of soma and nucleus was also measured. Again, the PNP1^−/−^ and PNP2^−/−^ neurons had significantly reduced soma (p < 0.001) and nuclei volumes (p < 0.001) in comparison to CTL1 and CTL2 neurons (Fig. 1B, C). To analyze potential effects on synapse formation, the number of axon terminals making contact with the soma or dendrites was analyzed. PNP1^−/−^ neurons had on average 1.47 ± 0.99 contacts, which was not significantly different than the 1.67 ± 1.07 contacts observed with the CTL1 neurons (p = 0.20, n = 30 neurons per replicate, 3 replicates). The differentiation of NPCs also yields glial progenitor cells capable of giving rise to astrocytes and oligodendrocytes. The percentage composition of glial progenitors in culture was compared, revealing no significant differences, with PNP1^−/−^ iPSCs generating 11.92% ± 4.04% and CTL1 iPSCs generating 12.65% ± 3.85% (p = 0.083, n = 10 images per replicate, 3 replicates), suggesting that PNP deficiency does not affect the relative composition of neurons in culture.

### Increased apoptosis in PNP-deficient neurons

**Figure 2:**
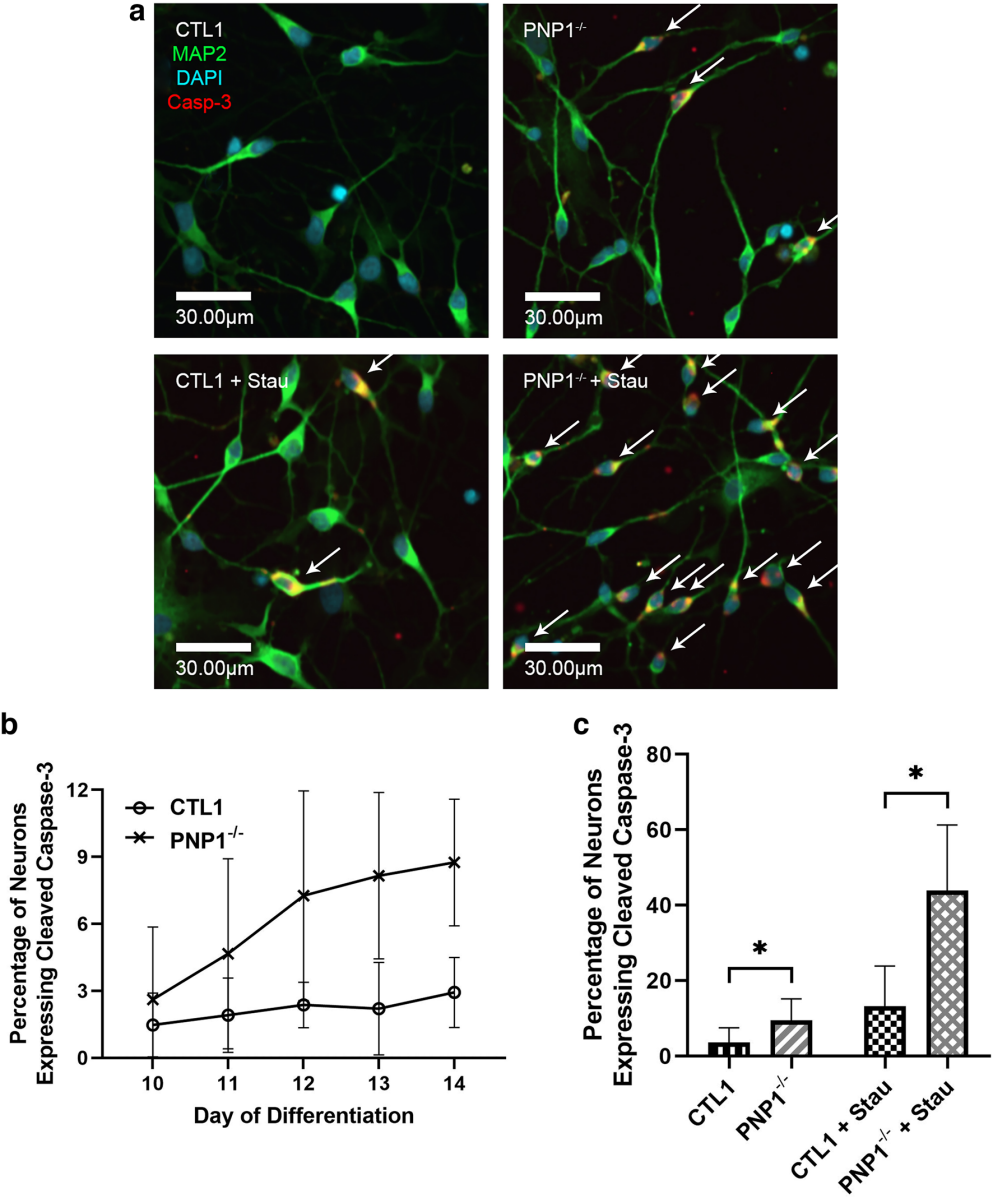
Increased spontaneous and induced cleaved caspase-3 expression in PNP-deficient induced pluripotent stem cell-derived neurons. (**A**) Representative immunofluorescence microscopy images depicting the presence of cleaved caspase-3 (casp-3) in neurons derived from control (CTL) and PNP-deficient (PNP^−/−^) iPSCs with and without staurosporine (stau). White arrows designate neurons expressing casp-3. (**B**) The average percentage of neurons exhibiting casp-3 expression from CTL1 and PNP1^−/−^ neurons across days 10–14 of differentiation to depict the rate of apoptosis. (**C**) The average percentage of neurons exhibiting casp-3 expression from CTL1 and PNP1^−/−^ neurons with and without stau on day 14. Data are the mean + SD of n = 90 (30 neurons from 3 replicates) for apoptotic rate, and n = 1050 (350 neurons from 3 replicates) for spontaneous and induced apoptosis; * p < 0.001.

Increased apoptosis was previously observed in sections of PNP-KO mice brains^12^. To determine if a similar process was also occurring in human PNP-deficient neurons, the rate of spontaneous apoptosis was determined by immunofluorescent microscopy measurement of the expression of cleaved casp-3, a marker of apoptosis (Fig. 2A). The PNP1^−/−^neurons exhibited elevated spontaneous apoptosis across each day, as well as an increased rate of apoptosis in comparison to the CTL1 neurons (Fig. 2B). Spontaneous and stau-induced apoptosis on day 14 was also assessed (Fig. 2A), revealing a significantly increased percentage of PNP-deficient neurons expressing cleaved casp-3 at rest (p < 0.001) (Fig. 2C). Following treatment with stau, 43.93% ± 17.38% of PNP1^−/−^ cells expressed casp-3 in comparison to only 13.14% ± 10.75% of CTL1 (p < 0.001) (Fig. 2C), demonstrating the increased susceptibility of PNP-deficient cells to apoptosis.

### Increased intrinsic apoptosis of PNP-deficient neurons

**Figure 3:**
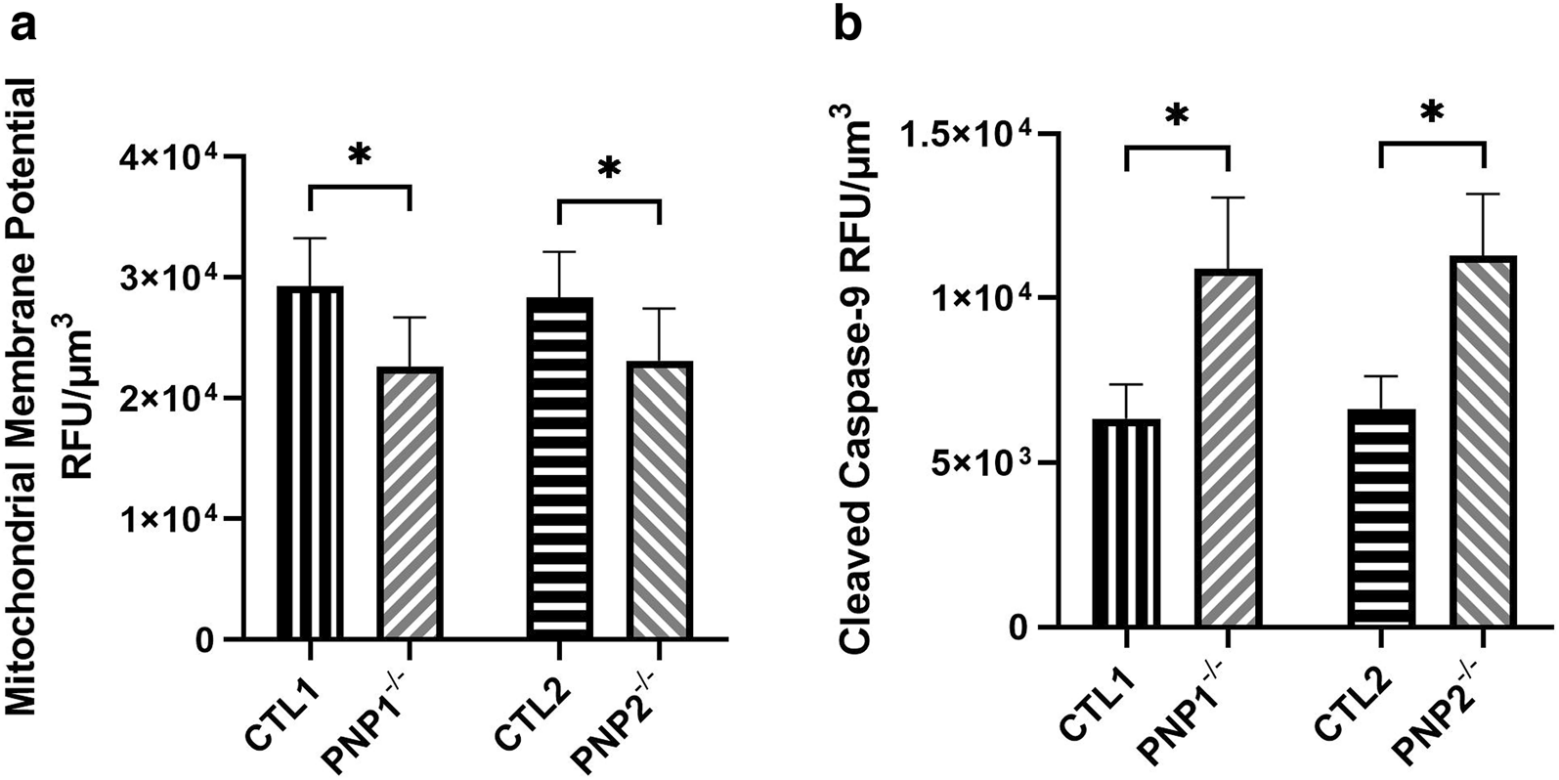
Elevated intrinsic apoptosis in PNP-deficient induced pluripotent stem cell-derived neurons. The mitochondrial membrane potential (**A**) and cleaved caspase-9 (**B**) fluorescence of neurons derived from control (CTL) and PNP-deficient (PNP^−/−^) iPSCs. The average relative fluorescence units (RFU)/µm^3^ was calculated by measuring fluorescence throughout the whole volume of a neuron. Data shown as mean + SD of n = 1050 (350 neurons from 3 replicates); * p < 0.001.

Previous studies have indicated that the increased apoptosis of PNP-deficient thymocytes is initiated in the mitochondria^3^. Therefore, MMP in iPSC-derived neurons was measured as an indicator for mitochondrial integrity and activation of the intrinsic apoptotic pathway. The fluorescence in sections of PNP1^−/−^ neurons was significantly lower (p < 0.001) than that of PNP-proficient neurons (Supplemental Fig. 2). Similarly, PNP2^−/−^ neurons exhibited a significant reduction in MMP fluorescence compared to CTL2 neurons (p < 0.001) (Supplemental Fig. 2). Again, to avoid potential bias when analyzing single focal planes, fluorescence was also assessed across entire cells. MMP fluorescence was significantly reduced (p < 0.001) in the PNP1^−/−^ and PNP2^−/−^ neurons compared to CTL1 and CTL2 (Fig. 3A). Furthermore, the fluorescence of the downstream intrinsic apoptotic protein cleaved casp-9 was significantly increased (p < 0.001) in PNP1^−/−^ and PNP2^−/−^ compared to CTL1 and CTL2 neurons, respectively (Fig. 3B). Together, the reduced MMP and increased cleaved casp-9 fluorescence indicate the engagement of the intrinsic apoptotic pathway in PNP-deficient neurons.

### Increased p53 causes abnormalities in PNP-deficient neurons

**Figure 4:**
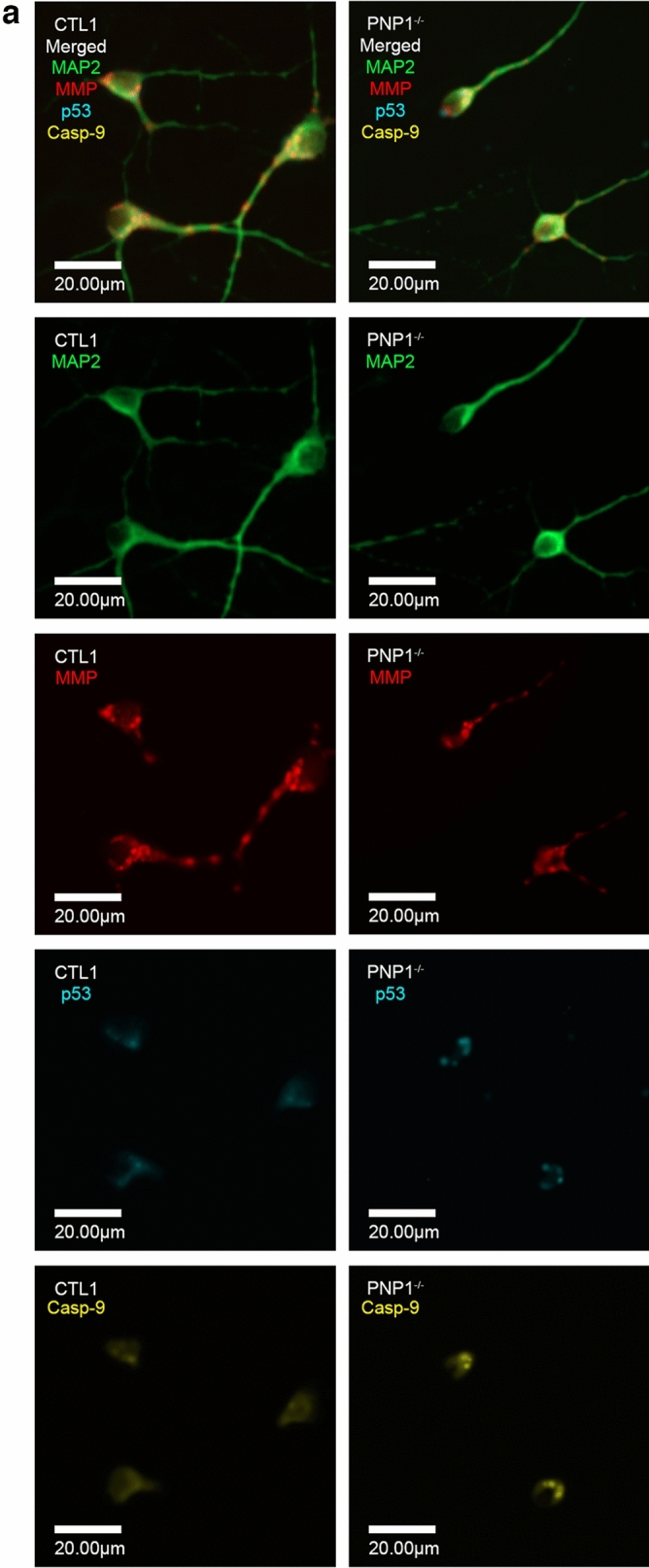

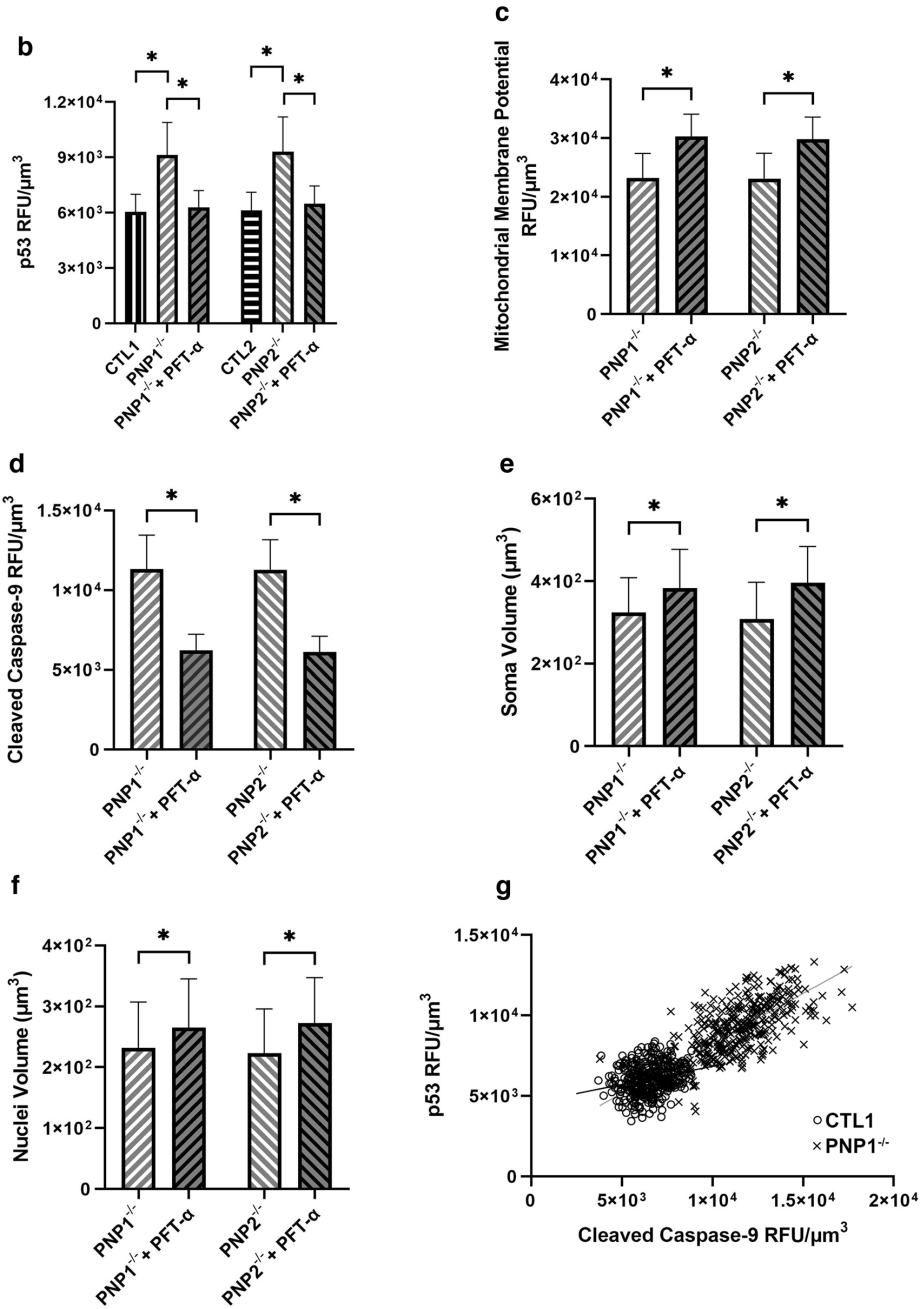
Inhibition of p53 with pifithrin-α restores healthy phenotype in PNP-deficient induced pluripotent stem cell-derived neurons. (**A**) Representative immunofluorescence microscopy images depicting the mitochondrial membrane potential (MMP), cleaved caspase-9 (casp-9), and p53 in neurons derived from control (CTL) and PNP-deficient (PNP^−/−^) iPSCs. The average fluorescence of p53 (**B**), MMP (**C**), and casp9 (**D**), and the average soma (**E**) and nuclei (**F**) volumes of neurons derived from control (CTL) iPSCs, and PNP-deficient (PNP^−/−^) iPSCs with or without p53 inhibitor pifithrin-α (PFT-α) supplemented. (**G**) The linear regression of casp-9 and p53 fluorescence of PNP1^−/−^ and CTL1. The average relative fluorescence units (RFU)/µm^3^ was calculated by measuring fluorescence throughout the whole volume of a neuron. Data shown as mean + SD of n = 1050 (350 neurons from 3 replicates); *p < 0.001.

The tumor suppressor protein p53 was previously implicated in mediating the apoptosis associated with abnormalities in purine metabolism, therefore p53 expression was assessed in PNP-deficient neurons (Fig. 4A). Significantly increased (p < 0.001) p53 fluorescence was detected in PNP-deficient neurons compared to PNP-proficient neurons (Fig. 4B). To further confirm the role of p53 in the apoptosis caused by PNP deficiency, PNP1^−/−^ and PNP2^−/−^ iPSCs were treated with PFT-α, a p53 transcription inhibitor. The addition of PFT-α to the PNP-deficient neurons normalized MMP (Fig. 4C) and cleaved casp-9 expression in these cells (Fig. 4D). Moreover, PFT-α treatment significantly increased (p < 0.001) the volume of the PNP-deficient neuronal soma and nuclei (Fig. 4E, F). In PNP^−/−^ cells, the R^2^ between p53 and cleaved casp-9 was much greater (p < 0.001, R^2^ = 0.5154) in comparison to PNP-proficient cells (p < 0.001, R^2^ = 0.0496) (Fig. 4G), suggesting that the cleaved casp-9 expression might be linked to p53 expression in PNP-deficient neurons. Together, this data implicates p53 as an important mediator of the intrinsic apoptosis caused by PNP deficiency.

### RNR inhibition

**Figure 5:**
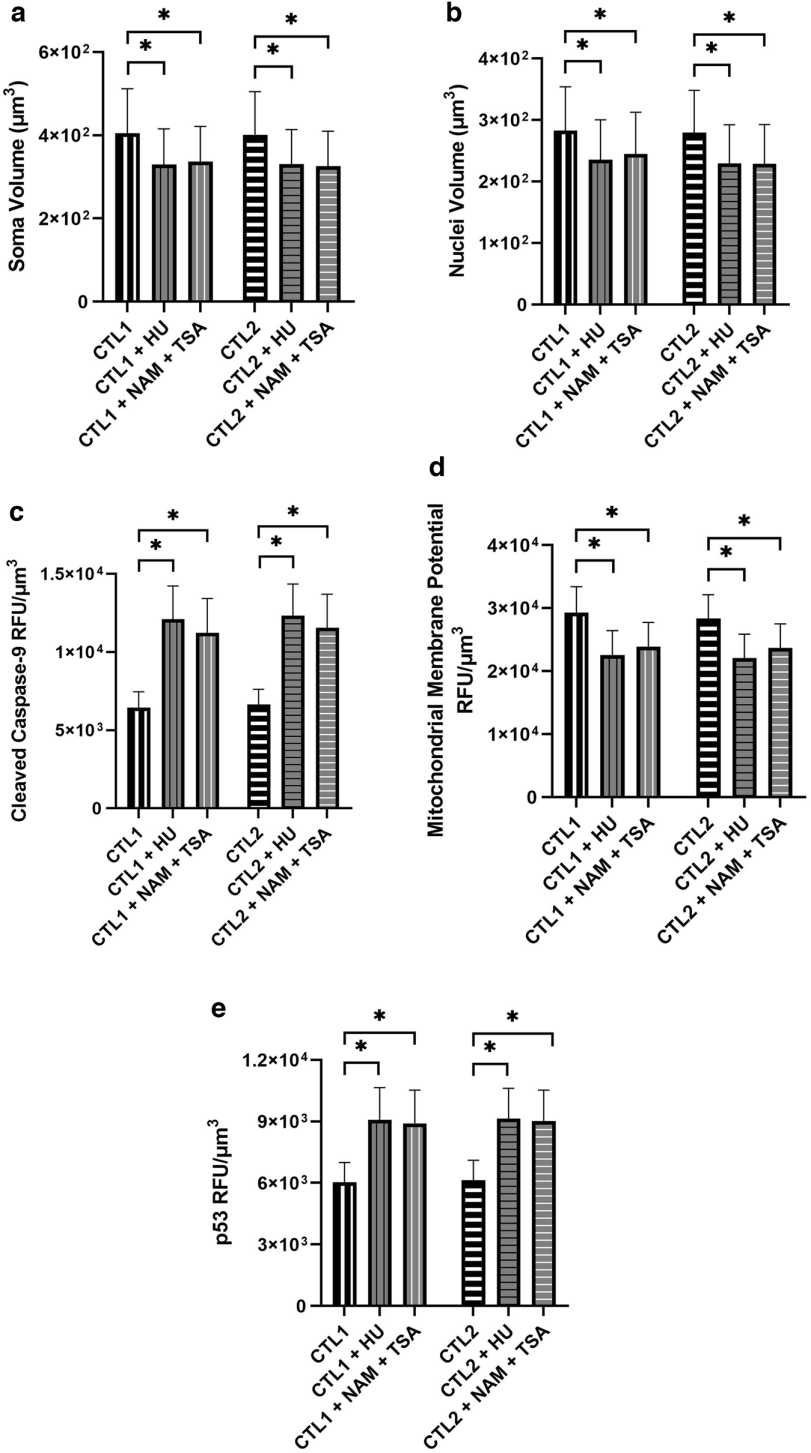
RNR inhibition recapitulates the phenotype of PNP-deficiency in healthy induced pluripotent stem cell-derived neurons. The soma (**A**) and nuclei (**B**) volume, the average mitochondrial membrane potential (**C**), cleaved caspase-9 (**D**), and p53 (**E**) fluorescence of neurons derived from control (CTL) iPSCs with or without RNR inhibitors hydroxyurea (HU) or nicotinamide (NAM) and trichostatin A (TSA) supplemented. The average relative fluorescence units (RFU)/µm^3^ was calculated by measuring fluorescence throughout the whole volume of a neuron. Data shown as mean + SD of n = 1050 (350 neurons from 3 replicates); *p < 0.001.

Inhibition of RNR in murine models causes neuronal abnormalities similar to those seen in the PNP-KO mice^7,13,14^, suggesting that RNR dysfunction might contribute to the intrinsic apoptosis observed in PNP-deficient neurons. Therefore, normal iPSC-derived neurons from CTL1 and CTL2 were treated with the RNR inhibitor HU, which led to a significant reduction in the average soma (p < 0.001) and nuclei (p < 0.001) volumes of the cells (Fig. 5A, B). Additionally, a significant (p < 0.001) reduction in MMP fluorescence (p < 0.001) (Fig. 5C), as well as a significant increase in the expression of cleaved casp-9 (p < 0.001) (Fig. 5D) and p53 (p < 0.001) (Fig. 5E) were observed. Similar effects on neuronal apoptosis were also seen with an alternative RNR inhibitor, the combination of NAM and TSA, that exert their effects by disrupting RNR homodimer assembly. Treatment of CTL1 and CTL2 neurons with NAM and TSA led to significant reductions (p < 0.001) in MMP fluorescence, as well as significant (p < 0.001) increases in cleaved casp-9 and p53 expression. Hence, RNR inhibition in PNP-proficient cells recapitulated all the abnormalities observed in PNP-deficient neurons, implicating RNR as a potential mediator of the effects of PNP deficiency.

### PNP supplementation can reverse the abnormalities of PNP-deficient neurons

**Figure 6:**
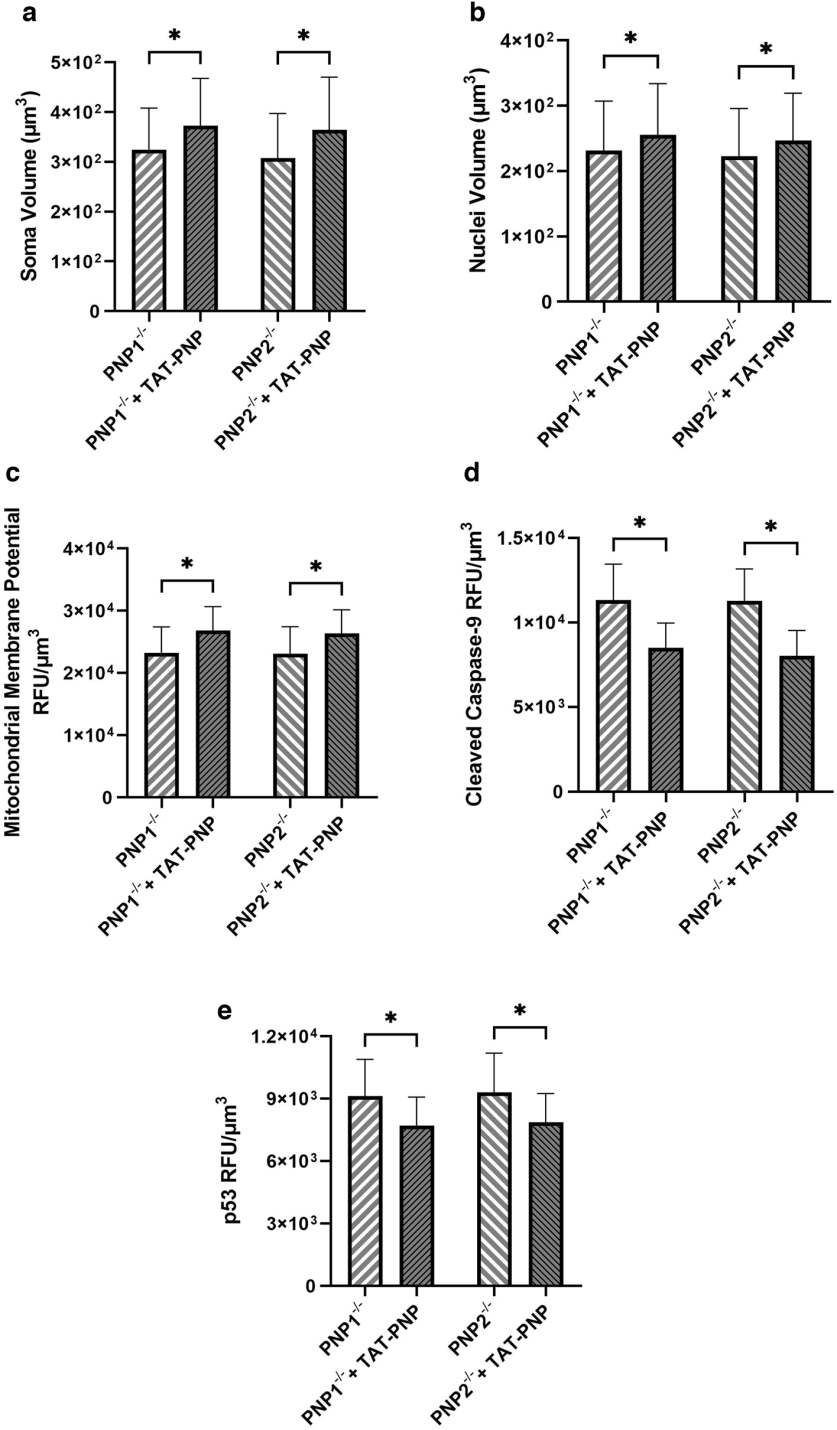
PNP supplementation reduced intrinsic apoptosis from PNP-deficient induced pluripotent stem cell-derived neurons. The soma (**A**) and nuclei (**B**) volume, the average mitochondrial membrane potential (**C**), cleaved caspase-9 (**D**), and p53 (**E**) fluorescence of neurons derived from PNP deficient (PNP^−/−^) iPSC-derived neurons with or without TAT-PNP replacement enzyme supplemented. The average relative fluorescence units (RFU)/µm^3^ was calculated by measuring fluorescence throughout the whole volume of a neuron. Data shown as mean + SD of n = 1050 (350 neurons from 3 replicates); *p < 0.001.

To confirm that the abnormal purine metabolism was the cause of the abnormalities observed in the PNP-deficient neurons and to test its ability to prevent these abnormalities, cells were treated with TAT-PNP, previously shown to restore PNP enzyme activity within cells^23^. PNP supplementation resulted in a significant increase (p < 0.001) in the average soma and nuclei volumes (p < 0.001) of PNP1^−/−^ and PNP2^−/−^ neurons compared to untreated cells (Fig. 6A,B), while CTL1 (p = 0.11) and CTL2 (p = 0.21) neurons were not significantly affected (data not shown). Following TAT-PNP treatment, MMP fluorescence of PNP-deficient neurons, measured along a single focal plane (Supplemental Fig. 3) and throughout the entire neuron (Fig. 6C), increased significantly (p < 0.001) while cleaved casp-9 expression decreased significantly (Fig. 6D). Similarly, p53 fluorescence was significantly reduced (p < 0.001) with TAT-PNP treatment (Fig. 6E), suggesting that a deficiency in PNP was responsible for the elevation in p53 expression. The PNP-proficient neurons exhibited no significant differences in MMP, cleaved casp-9, or p53 fluorescence (p > 0.05, data not shown), suggesting that PNP supplementation only affects PNP-deficient neurons.

## Discussion

Mice lacking PNP suffer from neuromotor abnormalities reminiscent of those affecting PNP-deficient patients^12^. However, the mechanism for the human manifestations has remained elusive, in part because of the ethical and technical inability to obtain brain tissue from patients. The availability of iPSC-derived cells has accelerated studies of tissues that were difficult to reach, such as neuronal cells. Our preliminary work previously identified that neurons generated from iPSCs derived from PNP-deficient patients had reduced soma and nuclei areas as well as enhanced spontaneous apoptosis^18^. Here significant alterations in the soma and nuclei volumes as well as increased spontaneous and stau-induced apoptosis in PNP-deficient neurons are demonstrated. The accelerated apoptosis of PNP-deficient neurons is concordant with the increased sensitivity to irradiation of PNP-deficient T lymphocytes and bone marrow cells^11^ as well as lymphoblastoid B cells that were previously demonstrated^26^. Hence, we hypothesize that in PNP-deficient patients, there is increased neuronal susceptibility to apoptosis. Moreover, PNP-deficient neurons had increased expression of activated cleaved casp-9, suggesting that the apoptosis of the cells is intrinsic, similar to the findings in thymocytes from PNP-deficient mice^2^. The finding of intrinsic mitochondrial-mediated apoptosis in PNP-deficient neurons may also support the notion that the predominant effects of the accumulation of dGuo and its derivative dGTP, observed with defects in PNP function, could be attributed to the localization of dGK in the mitochondria^3^.

The continuous availability of PNP-deficient neurons from iPSCs provided an opportunity to study the mechanisms leading to the enhanced intrinsic apoptosis of these cells. The apoptosis of the PNP-deficient neurons was associated with increased expression of p53, while inhibition of p53 prevented the morphological abnormalities and death of PNP-deficient cells, further implicating p53 as an important mediator of the intrinsic apoptosis caused by PNP deficiency. Interestingly, these results differ from reports that demonstrated that apoptosis induced by chemical inhibition of PNP in lymphocytes from patients with chronic lymphocytic leukemia occurred in a p53-independent manner^27^. The discrepancy in apoptosis pathways between the PNP-defective leukemic cells and the neuronal cells might be due to the differing rates of proliferation, although p53-mediated apoptosis was also demonstrated in the rapidly proliferating thymocytes from PNP-deficient mice^3^. Alternatively, the differences in the role p53 maintains in cell survival might be due to a cell-specific sensitivity, as previously demonstrated in hepatocellular carcinoma cell lines^28^.

Accumulation of dGTP has been proposed as a cause for allosteric inhibition of RNR^3^, and impaired RNR function in murine causes neuronal abnormalities like those observed in PNP-deficient mice^7,13,14^. Additionally, p53 is known to regulate RNR activity in response to DNA damage^29–31^, therefore the potential contribution of RNR alterations to the phenotype of PNP-deficient neurons was explored. Due to the absence of commercially available compounds to restore RNR function, the effects of RNR inhibition were investigated using two complementary approaches. HU can inhibit RNR by diffusing into cells and quenching the tyrosyl free radical at the active site of RNR thereby preventing the reduction of ribonucleotides. This has several downstream consequences, including depletion of the dNTP pool, induction of p53 expression and accumulation, and stalling the replication of cells at the G_1_/S phase; effects that culminate in the apoptosis of a variety of cells including neuroepithelial cells^6,7,32^. In addition, RNR function was disrupted in PNP-proficient iPSC-derived neurons using NAM and TSA, which directly interfere with RNR homodimer assembly^19^. Both strategies led to reduced soma and nuclei volumes as well as enhanced apoptosis, akin to the changes seen in PNP-deficient neurons, demonstrating that the abnormalities associated with defective PNP function represented impending apoptosis of the cells. The inhibition of RNR may also contribute to the increased rate of apoptosis observed in the PNP-deficient neurons. Neurons, for example, contain an increased number of mitochondria relative to other cell types due to their reliance on mitochondrial respiration to generate ATP necessary for signaling^33^. This in turn may result in greater oxidative species production as they mature, which has been shown to cause increased oxidative mitochondrial DNA damage in neurons^34^. The depleted dNTPs in PNP deficiency, as a consequence of RNR inhibition, might interfere with mitochondrial DNA repair, ultimately resulting in apoptosis. Alternatively, the steady accumulation of dGTP may trigger the initiation of apoptosis, causing even more dGTP to be released into the extracellular space from apoptotic cells, increasing the rate of apoptosis, as a similar mechanism is thought to propel the apoptosis of thymocytes^2^. Similar to the findings presented here on the role of RNR in the development of neuronal damage in PNP deficiency, RNR inhibition was recently shown to impair neutrophil differentiation in adenosine deaminase deficiency^35^, which also emphasizes the overlap between these two purine defects. Understanding the mechanism of neuronal cell damage might provide future avenues for directed anti-apoptotic interventions in PNP-deficient patients. In addition, appreciating the effects exerted by RNR inhibitors, such as HU, on neuronal cells might also explain the increased risk of neuropathy seen in patients with HIV^36^, a topic that was not studied here.

To conclusively demonstrate that the abnormalities observed in the PNP-deficient iPSC-derived neuronal cells were directly related to the abnormal intracellular purine metabolism, rather than a flaw in the establishment of the iPSC or the differentiation process, PNP activity was restored using TAT-PNP. Treating iPSC-derived PNP-deficient neurons with TAT-PNP improved their viability, prevented the morphological abnormalities, and halted the accelerated apoptosis, providing indisputable evidence for the effects of PNP deficiency on the cells. These findings also have important implications for the management of PNP-deficient patients. TAT-PNP was previously shown to correct intracellular PNP activity in lymphocytes^23^ and TAT-PNP treatment since birth prevented the neurological abnormalities observed in PNP-deficient mice^12^. However, it was not clear whether TAT-PNP benefits were due to a direct effect on neuronal cells or possibly secondary to the concomitant immune reconstitution^23^. Here, direct proof that PNP supplementation can prevent the apoptosis of PNP-deficient neurons is provided. Notably, TAT-PNP was added to the cultures at the iPSC stage and replenished frequently, while treatment of PNP-deficient patients is typically initiated only after birth. In utero, maternal PNP can remove excess dGuo from the circulation, averting the damage to the fetus tissues, yet after birth there is rapid accumulation of dGuo, interfering with RNR function. Within 12 h of stalling replication following dNTP pool depletion, double-stranded breaks can be detected^37^. Hence the data presented here also reiterates the need for rapid identification of PNP deficiency and restoration of the purine homeostasis in neonates to prevent irreversible neuronal damage^38^.

The current study has several limitations. Despite the role demonstrated here for PNP in neuronal survival, only 50% of PNP-deficient patients develop neuronal abnormalities. Some of the variability might be due to residual enzyme expression, as demonstrated recently in a family with partial PNP deficiency^26^. Differences in neurological phenotypes even among siblings with identical mutations, however, suggest that additional, and still unknown susceptibility factors might exist, such as expression of RNR, exposure to oxidative stress, etc. Additionally, the in vitro conditions and the non-specific neurons generated from iPSCs are unable to fully recapitulate the complex cell-to-cell interactions, neurotransmitters involvement, and many other factors present in the human brain. Specific staining for synapse formation or electrophysiological studies were also not performed, though there did not seem to be significant differences in the contact that PNP-deficient axons made with soma, which is in line with previous findings that inhibiting RNR in iPSC-derived neurons does not affect neurite outgrowth, including process length and branching^39^.

As the reduced and apoptotic cerebellar Purkinje cells observed in PNP-deficient mice would not be sufficient to explain the smaller cerebellum^12^, the goal of this study was to examine the potential mechanisms that might affect diverse types of neuronal cells rather than focus on Purkinje cells. While neuronal subtypes were not determined, others have previously demonstrated that the dominant neurons produced are glutamatergic, with significantly smaller percentages of GABAergic and dopaminergic neurons^40,41^. The identification of mitochondrial-mediated apoptosis in PNP-deficient neurons will now allow further focus on subtypes that might lead to preferential susceptibility, such as motor neurons. Indeed, upper motor neuron degeneration, which may contribute to the motor abnormalities in PNP deficiency, is characteristic of numerous neurodegenerative diseases, many with mitochondrial defects^42^. While the differentiation of NPCs also gave rise to glial cells^43^, preliminary investigations did not indicate a major role for PNP, as the percentage of glial progenitors from PNP-deficient iPSCs did not differ from healthy controls. Nevertheless, future experiments utilizing dedicated differentiation protocols to generate Purkinje cells^44^, motor neurons^45^, and mature glial cells^46^ such as astrocytes and oligodendrocytes, could further help understand the complex phenotype observed in PNP deficiency.

Ultimately, this study provides an important indicator that enhanced apoptosis is responsible for at least some of the neurological abnormalities affecting PNP-deficient patients and suggests treatment avenues to alleviate the phenotype. Also, the potential role of extracellular purine metabolites alterations, such as dGuo, Guo and their derivatives, on neuronal developments was not assessed, as the iPSCs and the derived cells require frequent replacement of culture media, thereby removing toxic substances that might accumulate in vivo. Additional contributing factors to neuronal apoptosis, such as increased oxidative stress, were not explored^47^ and will need to be addressed in future studies.

In conclusion, we demonstrate here the essential role of PNP in the development of human neurons and emphasize the importance of better understanding and treating neuronal damage in PNP-deficient patients.

## Supplementary Information

Supplementary Information.

## Data Availability

All datasets generated for this study are included in the article/supplementary material.

## Abbreviations

Casp-3: Caspase-3
Casp-9: Caspase-9
CTL: Control
dGK: Deoxyguanosine kinase
dGuo: Deoxyguanosine
dGTP: Deoxyguanosine triphosphate
dNTP: Deoxynucleotide triphosphate
EB: Embryoid body
Guo: Guanosine
HU: Hydroxyurea
iPSC: Induced pluripotent stem cell
MMP: Mitochondrial membrane potential
NAM: Nicotinamide
NPC: Neural Progenitor Cell
PFT-α: Pifithrin-α
PNP: Purine nucleoside phosphorylase
RNR: Ribonucleotide reductase
PNP-KO: PNP-knockout
RFU: Relative fluorescence units
Stau: Staurosporine
TAT-PNP: Purine nucleoside phosphorylase conjugated with trans-activator of transcription
TSA: Trichostatin A
