# Supplementary material for: Purine nucleoside phosphorylase deficiency induces p53-mediated intrinsic apoptosis in human induced pluripotent stem cell-derived neurons
Source: Sci Rep. 2022 May 31;12:9084. doi: 10.1038/s41598-022-10935-0 (PMC9156781; doi:10.1038/s41598-022-10935-0)
Supplement: Supplementary file 1 — Supplementary Information. [file 41598_2022_10935_MOESM1_ESM.docx]

**Supplemental Figures**


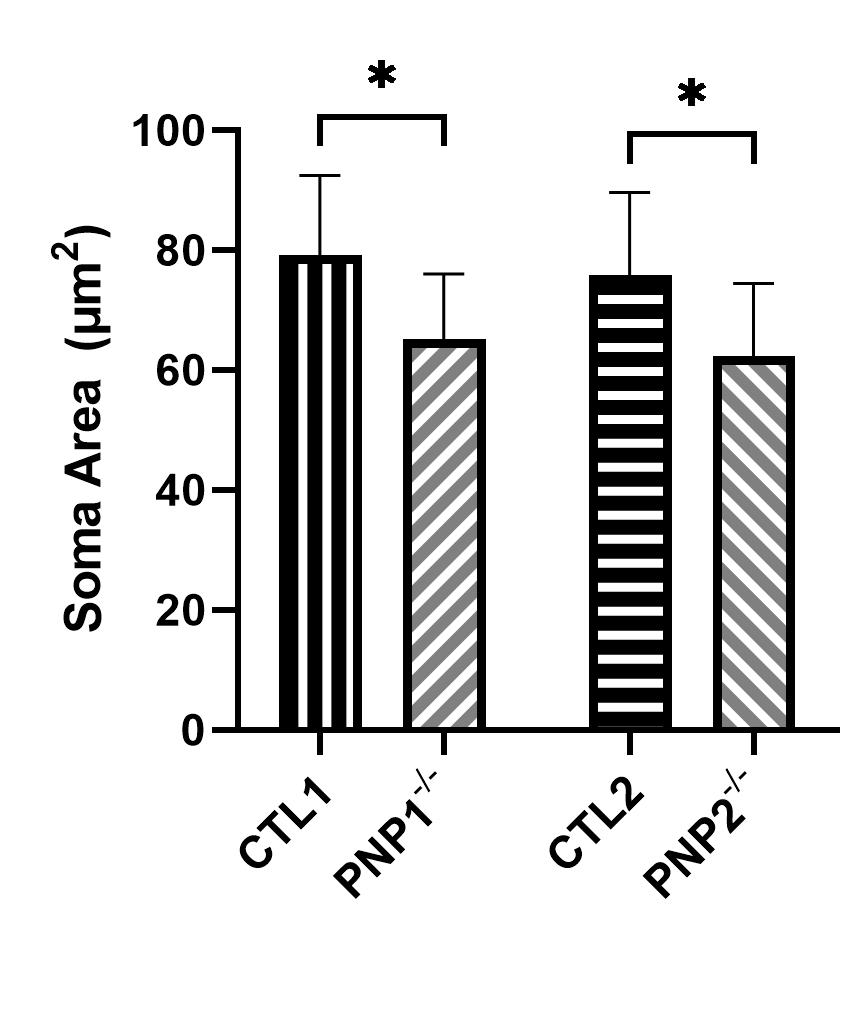

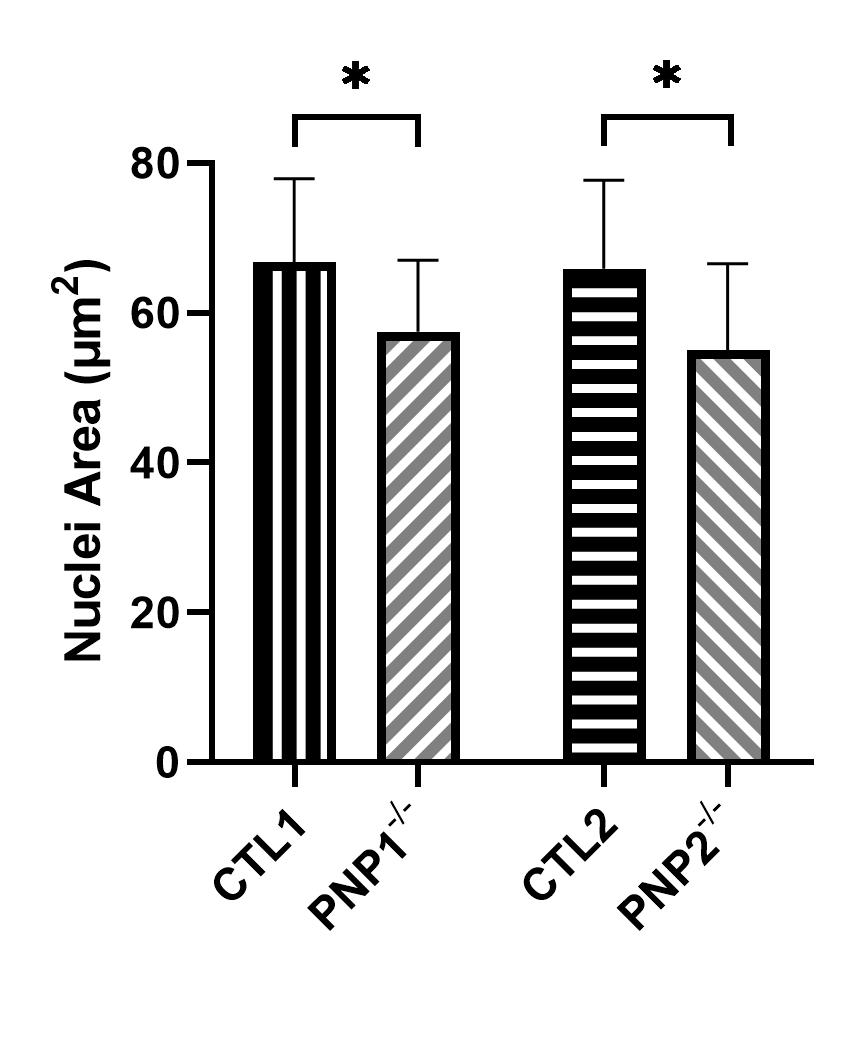


A

B

Supplemental Figure 1: Reduced soma and nuclei area from PNP-deficient induced pluripotent stem cell-derived neurons.

The average soma (A) and nuclei (B) areas of neurons derived from control (CTL) and PNP-deficient (PNP^-/-^) iPSCs. The average area was calculated by measuring across a single focal plane of a neuron. Data are the mean + SD of n=1050 (350 neurons from 3 replicates); * p<0.001.


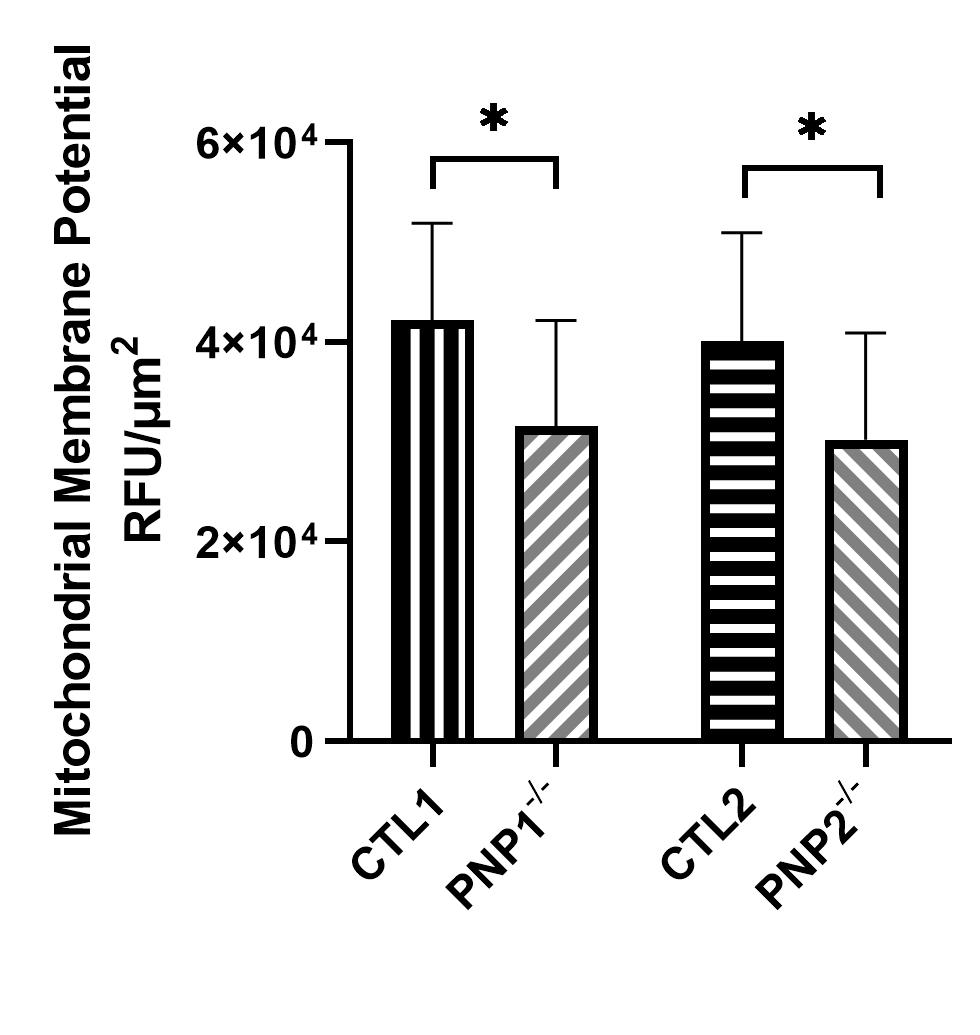


Supplemental Figure 2: Reduced mitochondrial membrane potential in PNP-deficient induced pluripotent stem cell-derived neurons.

The mitochondrial membrane potential (MMP) fluorescence of neurons derived from control (CTL) and PNP-deficient (PNP^-/-^) iPSCs. The average relative fluorescence units (RFU)/µm^2^ was calculated by measuring fluorescence across a single focal plane of a neuron. Data shown as mean + SD of n=1050 (350 neurons from 3 replicates); * p<0.001.


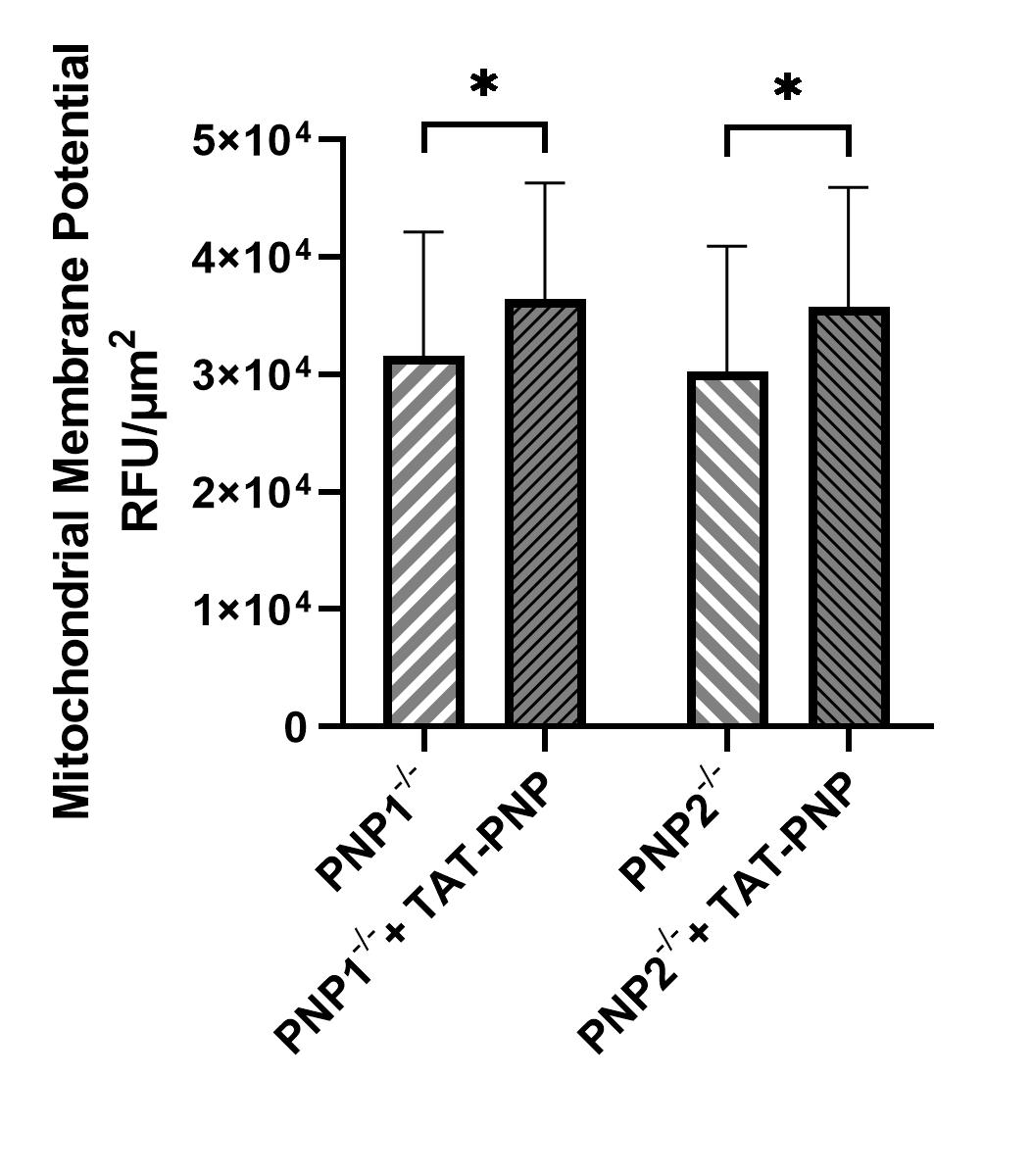


Supplemental Figure 3: PNP supplementation increased mitochondrial membrane potential in PNP-deficient induced pluripotent stem cell-derived neurons.

The mitochondrial membrane potential (MMP) fluorescence of neurons derived from PNP-deficient (PNP^-/-^) iPSCs with or without TAT-PNP replacement enzyme supplemented. The average relative fluorescence units (RFU)/µm^2^ was calculated by measuring fluorescence across a single focal plane of a neuron. Data shown as mean + SD of n=1050 (350 neurons from 3 replicates); * p<0.001.
